# Hepatic Dearterialization for Nonresectable Liver Tumors in Five Dogs and Two Cats

**DOI:** 10.1111/jvim.70023

**Published:** 2025-03-12

**Authors:** Michelle T. Nguyen, Chick Weisse, Stacy Kaneko

**Affiliations:** ^1^ Schwarzman Animal Medical Center New York New York USA; ^2^ Cornell University College of Veterinary Medicine Ithaca New York USA

**Keywords:** carcinoma, coil embolization, interventional, liver tumor

## Abstract

**Introduction:**

Some massive or nodular liver tumors can make surgical resection dangerous. Transarterial embolization and chemoembolization recently have been evaluated in dogs and cats, but multinodular or diffuse tumors make selective embolization difficult, impractical, and may require multiple anesthetic events. Hepatic dearterialization in humans has been shown to be safe and sometimes successful in promoting temporary tumor regression.

**Materials and Methods:**

Retrospective review of patients with nodular, diffuse, or non‐resectable massive liver tumors that underwent transarterial coil embolization of the hepatic artery from the origin of the gastroduodenal artery to the proximal hepatic artery was performed. Data recorded included patient signalment, clinical signs, serum biochemical changes, cross‐sectional imaging results, complications, and response to treatment.

**Results:**

Seven patients (five dogs and two cats) underwent transarterial hepatic dearterialization and were included. All patients had increased pretreatment hepatocellular enzyme activities 24 h after surgery. All patients survived to discharge and five were discharged within 24 h after treatment. Two patients experienced mild short‐term vomiting and anorexia, one of which required repeat hospitalization. Repeat laboratory testing approximately 6 weeks after treatment indicated decreased ALT and AST activities in 5/6 and 4/5 patients, respectively. Repeat imaging identified tumor regression in 3/4 patients evaluated by computed tomography (CT). Survival time ranged from 50 to 505 days.

**Conclusion:**

Hepatic dearterialization should be further investigated as a palliative management option for multinodular and diffuse liver tumors because it may provide a minimally invasive, safe, and palliative option based on the observation that all patients survived to discharge and tumor regression was noted in three animals.

**Abbreviations**
ALP: alkaline phosphatase
ALT: alanine aminotransferase
AMC: Animal Medical Center
AST: aspartate transaminase
CBH: chronic bridging hepatitis
CR: complete remission
CT: computed tomography
CTA: computed tomography angiogram
DexSP: dexamethasone sodium phosphate
DM: diabetes mellitus
DOA: dead‐on‐arrival
GDA: gastroduodenal artery
GGT: gamma‐glutamyl transferase
HA: hepatic artery
HCC: hepatocellular carcinoma
HCT: hematocrit
ICU: intensive care unit
IR: interventional radiology
PD: progressive disease
PES: after‐embolization syndrome
PR: partial remission
RBA: resting bile acids
RI: reference interval
SD: stable disease
SS: stromal sarcoma
TACE: transarterial chemoembolization
TAE: transarterial embolization

## 1 Introduction

Primary hepatic masses are reported to account for approximately 0.6%–2.9% of tumors in dogs and cats [1, 2]. The most common of these, hepatocellular carcinoma (HCC), often is characterized as massive, nodular or diffuse [3, 4]. Complete resection is the preferred treatment for large solitary liver masses [1], but diffuse and nodular masses as well as massive liver tumors may not be amenable to safe, complete resection because of size and location. In addition to systemic chemotherapy and radiation therapy, other palliative treatment options such as transarterial embolization (TAE) and transarterial chemoembolization (TACE) have been investigated [5, 6].

The hepatic artery (HA) has been noted to be the primary arterial blood supply of liver tumors in humans and animals [7, 8]. As a result, HA ligation has been investigated as a treatment option for nonresectable hepatic tumors in people [9]. Open surgical hepatic arterial ligation was reported to be successful in managing primary and secondary liver tumors in humans [10] after noting successful dearterialization of rat tumors [11].

Transarterial coil embolization of the HA as a palliative treatment for non‐resectable liver masses has not been reported in animals with naturally occurring liver cancer and may be a safe alternative for patients with limited treatment options. We hypothesized that HA coil embolization would be safely tolerated in dogs and cats and could provide temporary tumor regression in some cases.

## 2 Materials and Methods

### 2.1 Criteria for Selection of Cases

A retrospective review was conducted of medical records of dogs and cats evaluated by the Schwarzman Animal Medical Center (AMC) interventional radiology (IR) service between 2018 and 2022 for non‐resectable massive, diffuse, or nodular hepatic masses. Patients were included if the patient underwent transarterial hepatic dearterialization procedure and had the following diagnostic and laboratory data: preoperative computed tomography (CT) performed ≤ 1 month before the procedure, laboratory diagnostic testing that included liver function tests and a complete blood count (CBC) performed 24 h before the procedure, 24 h after the procedure, and repeat laboratory diagnostic testing performed at least 6–12 weeks after the procedure in surviving patients.

### 2.2 Medical Records Review

Signalment, physical examination findings, previous treatments and management, biochemical test results, diagnostic imaging findings, and surgical and pathology reports were extracted from the medical records of cases included in the study.

### 2.3 Imaging

All pre‐ and postoperative tumor volumes were calculated using an image computing platform (3D Slicer, Mimics software, Materialize, Leuven, Belgium) on multiphase CT angiography (CTA) of normal liver and the liver tumors. Using Response Evaluation Criteria in Solid Tumors (RECIST) criteria [12], tumor response was categorized as complete remission (CR), partial remission (PR), stable disease (SD), or progressive disease (PD) based on tumor volume 6 weeks postoperatively in comparison to volumes calculated before the procedure. Complete remission was recorded when the presence of the tumor could no longer be identified. Partial remission was recorded if a decrease in volume of at least 30% was appreciated. Progressive disease was recorded when progressive volume of at least 20% was calculated. Finally, SD was considered in patients where tumor volumes had neither increased by 20% nor decreased by 30%.

### 2.4 Hepatic Artery Coil Embolization (Dearterialization Procedure)

**FIGURE 1:**
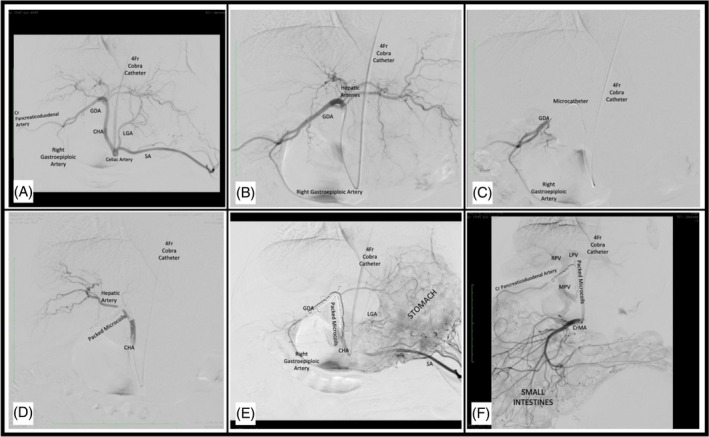
(A, B) The common hepatic artery (CHA) is accessed under fluoroscopic guidance using a 4 Fr angiographic catheter. (C, D) A microcatheter and micro‐wire combination were advanced into the gastroduodenal artery (GDA) and embolization microcoils were sequentially placed from the proximal GDA into the CHA, terminating just distal to the origins of the left gastric and splenic arteries. (E, F) Repeat angiography and cranial mesenteric arteriography confirms absent hepatic arterial flow and confirms occlusion of the GDA without retrograde arterial filling of the hepatic arteries. Abbreviations: CrMA, cranial mesenteric artery; LGA, left gastric artery; LPV, left portal vein; MPV, main portal vein; RPV, right portal vein; SA, splenic artery.

Vascular access was achieved through a 2–3 cm incision and surgical isolation of the right carotid artery. A 5 Fr vascular introducer sheath was secured for the duration of the procedure. The HA was accessed under fluoroscopic guidance using a 4 Fr angiographic catheter (Performa Cobra; Merit Medical, South Jordan, UT) and 0.035″ angled hydrophilic guidewire (Weasel Wire; Infiniti Medical, Redwood City, CA) combination. Digital subtraction angiography was performed to define the hepatic arterial anatomy via a celiac or common HA angiogram. A coaxially placed 2.4–2.8 Fr microcatheter (Merit Maestro; Merit Medical, South Jordan, UT) and micro‐wire combination were advanced into the gastroduodenal artery (GDA) and a variable number of various‐sized embolization microcoils was sequentially placed and tightly packed to embolize the proximal GDA down to the common HA, terminating just distal to the origins of the left gastric and splenic arteries in all animals. Absent hepatic arterial flow was confirmed by repeat angiography with evidence of contrast reflux into the celiac artery. Cranial mesenteric arteriography then was performed to confirm occlusion of the GDA and absence of retrograde arterial filling of the hepatic arteries. The catheters then were removed, the carotid artery ligated, and the surgical site closed routinely. An example of the procedure is shown in Figure 1.

### 2.5 Postoperative Care

Repeat CBC and serum biochemistry panels were performed approximately 24 h postoperatively. Repeat diagnostic imaging and laboratory diagnostic evaluation were recommended 6 weeks postoperatively. Follow‐up evaluation was done primarily through the IR service or by contact with the client or referring veterinarian when necessary.

## 3 Results

### 3.1 Signalment

Seven patients were evaluated by the AMC IR service for further management of nodular (five dogs) or massive (two cats) liver tumors that satisfied the study inclusion criteria (Table S3). Three female dogs and two male dogs (two Maltese mix, one Chihuahua mix, one Poodle mix and one Hound) ranged in age from 9 to 13 years old and weighed between 2.4 and 31.9 kg. In addition, a 5.2 kg, 12‐year‐old male neutered domestic longhair cat and a 4.7 kg, 13‐year‐old male neutered domestic shorthair cat were included.

### 3.2 Preoperative Findings

#### 3.2.1 History and Preoperative Physical Examination Findings (Table S4)

All animals were referred for nonresectable hepatic masses diagnosed by a referring veterinarian before evaluation by the IR service using preoperative diagnostic imaging (7/7) with or without surgical exploration (3/7). Three patients (3/7) had diagnosed heart disease on initial presentation; Patients #1 and #3 were diagnosed with degenerative mitral valve disease whereas one cat (Patient #7) was noted to have restrictive cardiomyopathy. At the time of presentation, three patients were noted to have cavitary effusions; two were dogs (Patients #1 and #2) with abdominal effusions suspected to be secondary to portal hypertension or tumor hypertension. One cat (Patient #7) had bi‐cavitary effusion that was suspected to be secondary to congestive heart failure and hemoabdomen secondary to the large hepatic mass. One dog (Patient #4) was suspected to have metastatic disease associated with a stromal sarcoma (SS) originally located in the spleen, which had been removed previously. One dog (Patient #5) was noted to have a 3‐year history of managed chronic bridging hepatitis (CBH) before the incidental finding of a large non‐resectable hepatic mass. Another dog (Patient #2) had a history of diabetes mellitus (DM) that was poorly regulated at the time of the procedure. Finally, two patients had gastrointestinal clinical signs manifested as diarrhea and hyporexia in a dog (Patient #3) and melena in a cat (Patient #7; Table S4).

One dog (Patient #3) had undergone hepatic TACE 4 months earlier. The owners elected to proceed with hepatic dearterialization when tumor progression was noted 3 months postoperatively.

#### 3.2.2 Preoperative Diagnostic Testing

All seven patients had preoperative abdominal CTA ≤ 4 weeks before the procedure. All dogs had multiple nodules throughout the liver, whereas both cats had solitary massive, non‐resectable hepatic masses. Five patients (three dogs and two cats) had histopathology or cytology performed. Tumor histopathology or cytology was obtained by incisional biopsy (2/5), punch biopsy (1/5), laparoscopic liver biopsy (1/5), or fine needle aspirate (1/5). Histopathology or cytology (Table S3) indicated HCC (1/5), SS (1/5), or a benign process (3/5), that was reported as a biliary cystadenoma (2/3) or inflammatory in nature (1/3). The patient diagnosed with an SS had evidence of metastatic disease on CT characterized as multiple pulmonary nodules measuring up to 5.4 mm in diameter and an aggressive polyostotic bone lesion affecting the vertebrae, consistent with metastasis. None of the patients had evidence of portosystemic shunting on CTA.

All seven patients had laboratory testing performed ≤ 24 h before the procedure that documented increases in hepatocellular enzyme activities above the reference interval (RI; Table S1). All dogs had increases in alanine aminotransferase (ALT), alkaline phosphatase (ALP), and gamma‐glutamyl transferase (GGT) activities. Four dogs had increased aspartate transaminase (AST) activity and one dog (Patient #4) with metastatic SS had normal AST activity of 50 U/L (RI, 16–55 U/L). All dogs had moderate to markedly increased ALT activity between 180 and 2067 U/L (RI, 18–121 U/L), variable increases in ALP activity ranging from 216 to 2926 U/L (RI, 5–160 U/L), and mild to markedly increased GGT activity ranging from 20 to 306 U/L (RI, 0–13 U/L). All dogs had preoperative resting bile acid (RBAs) concentrations measured, with mild to marked increases ranging from 17.2 to 166.4 μmol/L (RI, 0–14.9 μmol/L). Total bilirubin concentrations were within normal limits in all dogs (RI, 0–0.3 mg/dL).

Both cats (Patients #6 and #7) had similar testing performed preoperatively and were noted to have similar findings. Moderate increases in ALT at 284 and 454 U/L (RI, 27–158 U/L) and AST at 107 and 132 U/L (RI, 16‐67 U/L) were observed. Activity of GGT was mildly increased at 8 and 10 U/L (RI, 0–6 U/L). Unlike the dogs, the cats had normal ALP activities of 27 and 51 U/L (RI, 12–59 U/L). Patient #7 had increased total bilirubin concentration (4.2 mg/dL; RI, 0.0–0.3 mg/dL). Patient #6 had normal total bilirubin (0.1 mg/dL) and RBA of 5.3 μmol/L (RI, 0–6.9 μmol/L).

Complete blood counts (Table S2) were performed in all patients. Four dogs had a normal hematocrit (HCT) preoperatively; one dog (Patient #2) with DM was anemic (30.9%; RI, 38.3%–56.5%) with neutrophilia, lymphopenia, and leukocytosis. The four other dogs had normal leukocyte counts. One cat (Patient #6) had a normal hemogram. The other cat (Patient #7) was anemic (14.4%; RI, 28.2%–52.7%) with a moderate neutrophilic leukocytosis.

#### 3.2.3 Preoperative Medications

Four dogs and one cat were hospitalized 24 h before the procedure and received preoperative medications for ≤ 24 h before hepatic dearterialization. Preoperative antibiotics were given as either 30 mg/kg ampicillin/sulbactam (Unasyn, Pfizer, New York, NY) IV q8h in three dogs, a combination of ampicillin/sulbactam and 5–13 mg/kg metronidazole (Hospira; Lake Forest, IL) IV q12h in one dog, or 30 mg/kg cefoxitin iv q6h in one cat. Two patients (one dog and one cat) received preoperative steroids as 0.1 mg/kg dexamethasone sodium phosphate (DexSP; Bimeda‐MTC Animal Health Inc., Cambridge, ON, Canada) iv once.

Before induction, all patients received antiemetics either as 1 mg/kg maropitant citrate (Cerenia, Zoetis, Parsippany‐Troy Hills, NJ) iv q24h in one cat, 0.5 mg/kg ondansetron (Hikma; Berkeley Heights, NJ) iv q24h in one dog, or a combination of both in five dogs and one cat. In addition, two dogs received pantoprazole (Fresenius Kabi, Lake Zurich, IL) at 0.7 mg/kg intravenously.

### 3.3 Perioperative Findings

#### 3.3.1 Perioperative Medications

All patients received antibiotics under anesthesia as either 20–30 mg/kg cefoxitin (Fresenius Kabi USA LLC; Lake Zurich, IL) iv in three dogs and two cats, or a combination of both cefoxitin and 22 mg/kg ampicillin/sulbactam iv in two dogs. The remaining five patients that did not receive preoperative corticosteroids received an injection of dexamethasone at the time of extubation.

#### 3.3.2 Hepatic Artery Coil Embolization

**FIGURE 2:**
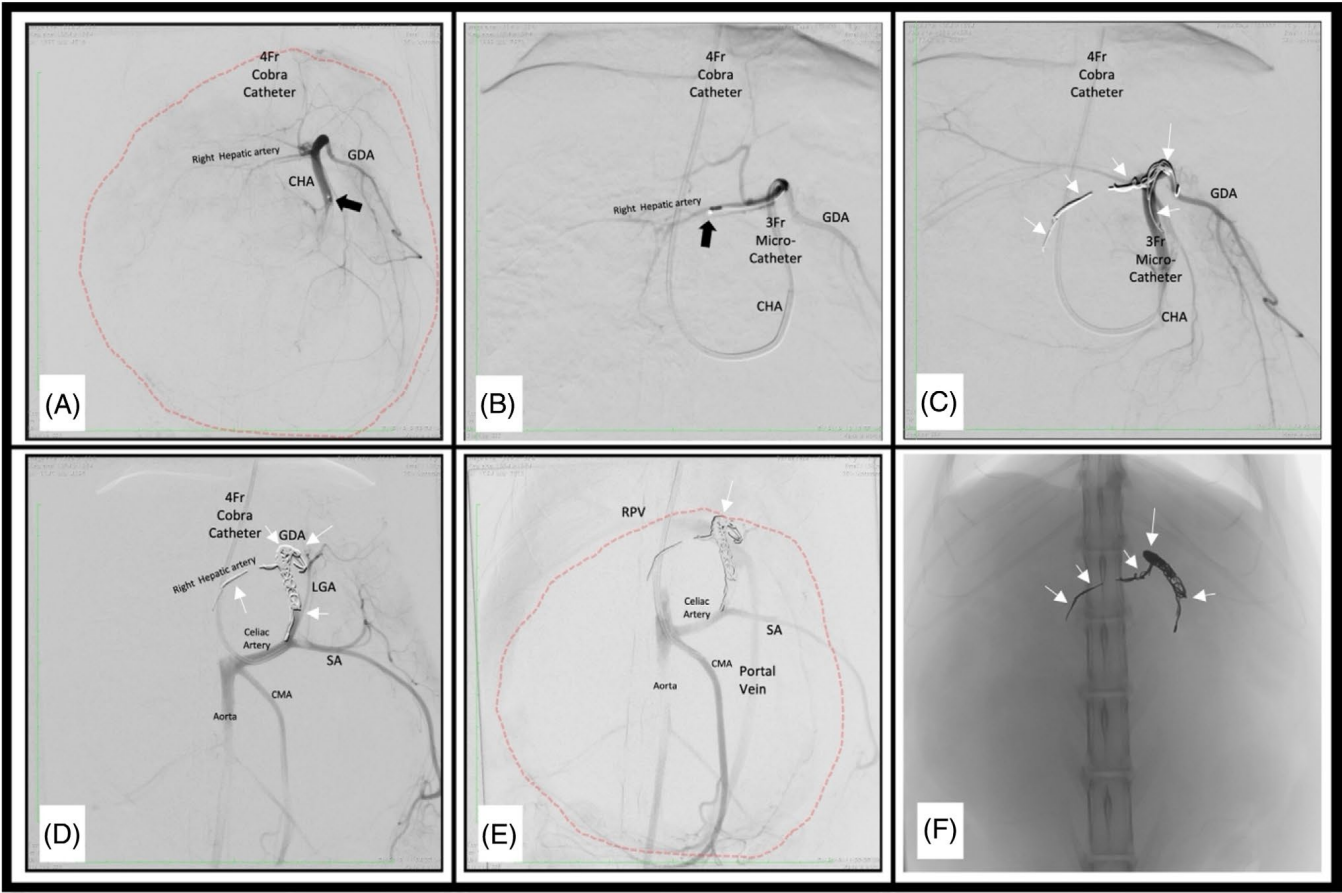
(A) Patient # 6 had an angiogram performed to outline the right hepatic artery, gastroduodenal artery (GDA) and the common hepatic artery (CHA). The catheter tip (black arrow) can be observed advancing into the selected arteries. The hepatic mass is appreciated (red dotted line). (B) A 3 Fr micro‐catheter is advanced and 0.56 mL of 100 μm embolic beads administered in 6.44 mL of saline and 5 mL of iodinated contrast administered slowly prior to coil embolization into a branch off the GDA. (C–E) Coil embolization. (F) Completed coil embolization (white arrows point to coils). Abbreviations: CMA, cranial mesenteric artery; LGA, left gastric artery; RPV, right portal vein; SA, splenic artery.

Coil embolization was performed as described above. The type and number of embolization coils were not reported in all patients but when reported, were noted to vary among patients. In addition, both cats also received additional embolic bead embolization. A bolus of 0.56 mL of 100‐μm embolic beads (LC beads, Boston Scientific, Washington DC, USA) in 6.44 mL of saline and 5 mL of iodinated contrast was administered slowly into the hepatic mass via a branch from the GDA before coil embolization in Patient #6 (Figure 2). Patient #7 received 0.5 mL of 100‐μm beads combined with an unreported volume of iodinated contrast administered after deployment of three coils to protect the GDA and before five additional coils to occlude the common HA No complications were noted intra‐operatively as a response to the technique performed.

### 3.4 Immediate Postoperative Findings

#### 3.4.1 Postoperative Medications

Patients were hospitalized for a minimum of 24 h after the procedure in the Intensive care unit. Patients received iv fluid support (Plasmalyte at 40–60 mL/kg/day or lactated Ringers solution at 60–75 mL/kg/day) for at least 24 h, analgesics, gastroprotectants, and antibiotics. Postoperative analgesia was administered to all patients as needed either as 0.1–0.2 mg/kg methadone (Mylan Pharmaceuticals, Washington DC, USA) iv q6h or 0.015–0.025 mg/kg buprenorphine (PAR Pharmaceutical, Chestnut Ridge, NY) iv q6–8h. Antinausea medications and gastroprotectants administered included maropitant citrate, ondansetron, and pantoprazole. Antibiotics administered included ampicillin/sulbactam (22–30 mg/kg iv q8h) and metronidazole (7–15 mg/kg iv q12h).

All patients received a 10–14 days course of antibiotics consisting of either 13.75–18 mg/kg amoxicillin trihydrate/clavulanate potassium (Clavamox, Zoetis, Parsippany‐Troy Hills, NJ) po q12h (3/7) or a combination of amoxicillin trihydrate/clavulanate potassium and 7–14 mg/kg metronidazole (Abbott Laboratories, Chicago, IL) po q12h (4/7). Five patients were discharged with analgesics that consisted of 1–2 mg/kg codeine (West‐Ward Pharmaceutical Corp; Eatontown, NJ) po q12h (3/5), 8–12 mg/kg gabapentin (Zhejiang Yongtai Pharmaceutical Co Ltd., Linhai, Zhejiang, China) po q8–12h (1/5), or 0.015 mg/kg transmucosal buprenorphine q8–12h (1/5). All patients were prescribed antiemetics, which consisted of either 1–2 mg/kg maropitant citrate (2/7) po q24h or a combination of maropitant citrate and 0.5–1 mg/kg ondansetron po q8–12h (5/7). Three dogs were prescribed 1.0–1.5 mg/kg omeprazole (Merit Pharmaceutical, Los Angeles, CA) po q12h indefinitely. A tapering course of an anti‐inflammatory dose of corticosteroids was given either as po prednisone or prednisolone.

One cat (Patient #7) was in congestive heart failure before dearterialization and fluid therapy was limited. As a result, this cat received packed red blood cell transfusions and enteral water via an esophagostomy tube. All but two patients (5/7) continued to receive a tapering course of prednisone or prednisolone po over a 12–30 days period; one dog (Patient #2) with DM did not receive corticosteroids postoperatively. Another dog (Patient #5) with CBH had received corticosteroids long‐term. Therefore, corticosteroids were continued at the original dose.

#### 3.4.2 Postoperative Diagnostic Testing: (Table S1)

All patients had serum biochemistry performed 24 h after hepatic dearterialization and marked increases in ALT and AST activities were observed. The ALT activities ranged from 736 to 16 138 U/L, a median increase of 6.5 times the baseline result. The AST activity ranged from 624 to 8297 U/L, a median increase of 10 times the baseline result. The ALP activity was variably changed with three dogs experiencing a progressive increase in ALP activity, static ALP activity in one cat, and decreased ALP activity in two dogs and one cat. Finally, all patients had decreased GGT activity.

All patients had a CBC performed 24 h after hepatic dearterialization, which disclosed a mildly progressive anemia in all patients except for one dog (Patient #3). Mild‐to‐moderate neutrophilia was noted in three dogs and one cat. Mild‐to‐moderate lymphopenia was noted in four dogs. The dog with CBH did not have clinically relevant changes on the hemogram.

#### 3.4.3 Short‐Term Postoperative Complications

All patients were discharged 24 (5/7) to 48 h (2/7) after the procedure.

Three patients were reported to have short‐term postoperative complications suspected to be associated with post‐embolization syndrome (PES). These consisted of gastrointestinal tract signs and pain or lethargy within the first 7 days after the procedure. One dog (Patient #1) had intermittent abdominal pain while hospitalized but no pain was reported by the owner at follow‐up examination. Two dogs had diarrhea, anorexia, vomiting, or a combination of these. Of patients experiencing gastrointestinal signs, only one dog (Patient #3) required rehospitalization for an additional 24 h for the placement of an esophagostomy tube. This dog was noted to have had diarrhea and hyporexia before hepatic dearterialization. The owners reported the dog had a normal appetite after discharge.

### 3.5 Long‐Term Follow‐Up

Only six patients had documented follow‐up examination after discharge. The dog with DM had limited follow‐up information available. This dog died as a consequence of unmanaged diabetic ketoacidosis after discharge against medical advice 8 weeks after hepatic dearterialization.

#### 3.5.1 Long‐Term Follow‐Up Diagnostics: (Table S1)

Five patients (three dogs and two cats; 5/6) were noted to have a follow‐up examinations 6–8 weeks postoperatively, whereas one dog had a follow‐up examination 11 weeks postoperatively.

Both cats had serum biochemistry performed 6 weeks postoperatively that indicated improvement of ALT, AST, ALP, and GGT activities to within normal RI (Table S1). The dogs (4/6) had variable results 6–11 weeks postoperatively. The ALT activity was improved in three dogs, ranging between 376 and 663 U/L, whereas the dog with metastatic SS had a progressively increased ALT of 731 U/L. The AST activity was evaluated in three of the four dogs, with improved results in two patients (140 and 116 U/L). The dog with CBH had slightly increased AST activity (338 U/L). The ALP activity was progressively increased in three of the four dogs evaluated (621–4229 U/L), whereas the patient that underwent hepatic TACE had an improved ALP activity (273 U/L) 11 weeks postoperatively. Finally, GGT activity increased mildly in two dogs, decreased in the dog with the history of previous TACE, and did not change in the dog with a history of CBH.

In addition to postoperative laboratory testing, both patients (Patients #1 and #7) reported to have abdominal effusion before dearterialization had abdominal ultrasonography repeated 2–3 weeks postoperatively, which confirmed resolution of effusion.

#### 3.5.2 Tumor Volume Assessment: (Table S4)

**FIGURE 3:**
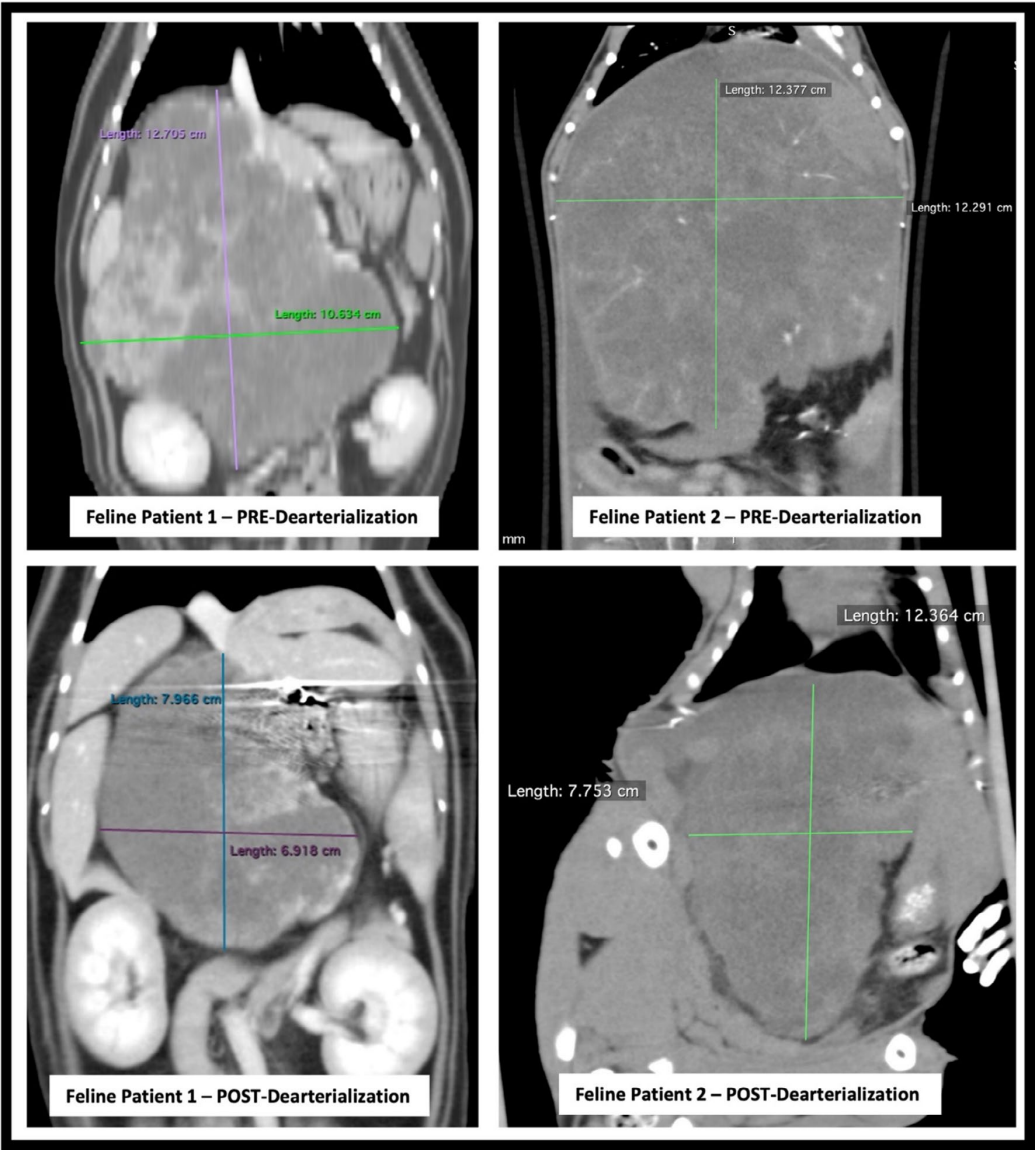
Feline Patient 1 (Patient # 6) was noted to have 63% reduction in tumor volume calculated by the 3D slicer program. The post‐dearterialization with contrast revealed no appreciable flow through the coils. Feline Patient 2 (Patient #7) was noted to have an overall 12% reduction in tumor volume. A post‐dearterialization angiogram was not performed and persistent flow could not be evaluated.

Four patients (two dogs and two cats) had tumor volumes reevaluated by repeated multiphase CTA (two dogs and one cat) or noncontrast CT (one cat). Tumor regression was noted in both cats and one dog. Median tumor regression was 12% in these three animals. One cat was reported to be in PR with a calculated tumor regression of approximately 63% (Figure 3). This patient's CTA verified persistent occlusion of the HA. The other cat had SD with 12% tumor regression. A CTA was not performed to evaluate revascularization. In two dogs with follow‐up CTA, vascular flow through the HA was visualized. One dog had SD with tumor regression of approximately 25% whereas the other dog had PD with tumor progression of approximately 45%.

One patient (Patient #3) did not undergo repeat CT evaluation and was euthanized 12 weeks postoperatively because of hemoabdomen. Necropsy volume measurements estimated tumor progression of approximately 27%. HA occlusion appeared to have persisted. This patient had developed hepatic hemangiosarcoma, which was not noted at the time of the procedure. The initial diagnosis was HCC based on prior incisional biopsy.

#### 3.5.3 Necropsy and Survival Time

Survival times ranged from 50 to 505 days. One patient was lost to follow‐up because of death associated with unmanaged diabetic ketoacidosis. Of the other six patients, four were euthanized and two dogs presented dead‐on‐arrival (DOA) to the hospital. One was DOA 119 days postoperatively secondary to congestive heart failure as confirmed on necropsy. The other dog with CBH was reported DOA by the referring veterinarian. A necropsy was not performed and the cause of death was unknown.

Of the four patients euthanized, one dog was euthanized because of decreased quality of life secondary to the previously diagnosed liver mass. The patient with metastatic SS was euthanized because of progressive spread of disease throughout the abdomen noted during abdominal exploratory surgery for a suspected gastrointestinal foreign body. One cat was euthanized 505 days after the procedure because of a decrease in quality of life secondary to hemoabdomen. A cause for the hemoabdomen was not determined. The other cat that previously received multiple blood transfusions was euthanized 220 days postoperatively because of decreased quality of life secondary to acute on chronic kidney disease.

## 4 Discussion

Ligation of the HA in dogs was once believed to be uniformly associated with death because of hepatic necrosis from anaerobic bacterial proliferation [13, 14]. Subsequent investigation indicated that preoperative treatment with antibiotics prevented bacterial proliferation after HA ligation, and recovery could be expected [13, 14, 15]. These studies concluded that the HA functioned to provide sufficient oxygenation to the liver in dogs to prevent anaerobic bacterial overgrowth [13, 14, 15]. All patients in our study received antibiotics as part of perioperative and postoperative treatment. This approach was used because of the previously‐mentioned studies confirming the requirement for prophylactic antibiotic administration to avoid anaerobic proliferation secondary to dearterialization of the liver [13, 14, 15, 16].

In patients undergoing transarterial coil embolization of the HA, minimal morbidity and no mortality was noted immediately postoperatively. Two had clinical signs potentially associated with PES. Post‐embolic syndrome is characterized by anorexia, vomiting, malaise and fever, or some come combination of these signs in humans [17]. Post‐embolic syndrome may be seen in approximately 45% of human patients who have undergone TACE [17]. In addition, PES was associated with a decrease in median survival times after TACE procedures in that study [17]. Little data is available regarding PES in dogs and cats after TAE or TACE of liver masses [6]. In a study involving drug‐eluting bead TACE in veterinary patients, 26% of procedures resulted in clinical signs associated with PES [14]. The authors reported no association between PES and procedural outcomes, nor was any pre‐ or intra‐procedural data predictive for the development of PES [6].

Immediately postoperatively, a presumed hypoxic event occurs with removal of the arterial supply to the liver, causing marked increases in ALT and AST activities in humans and animals [9, 18]. All dogs in a previous study survived immediately postoperatively and developed temporary marked increases in ALT and AST activities, with only one dog reported to acquire collateral vasculature secondary to incomplete dearterialization [15]. In addition, temporary hepatic embolization using gel foam led to hepatocellular damage noted by a marked increase in ALP activity that often resolved within 6 weeks [18]. Another study concluded that hepatic dearterialization did not affect hepatic excretory functions, and all changes to liver enzyme activities were transient and considered secondary to stress and ischemic injury [18]. The immediate postoperative laboratory diagnostic tests evaluated in our study were consistent with changes seen in previous studies [15, 18].

The activities of ALT and AST are known to increase with damage to hepatocytes [19]. Therefore, as liver tumors progress and outgrow local blood supply, these activities appear to increase secondary to presumed hepatocellular ischemia and subsequent necrosis [19, 20]. The improved results seen 6–11 weeks postoperatively in our study appear to coincide with tumor regression in both cats and 1 dog (Patient #1). Another study suggested that increased ALT and AST activities were associated with a worse prognosis for survival in patients with HCC [1]. Based on our small sample size, these results at reevaluation could not be evaluated as prognostic indicators. One dog (Patient #3) was not evaluated using advanced imaging postoperatively. However, postoperative laboratory testing 11 weeks after dearterialization indicated improved hepatic enzyme activities in comparison with preoperative results. Despite improved hepatic enzyme activities, necropsy performed the following week disclosed a progressive increase in liver tumor size. Necropsy evaluation of the hepatic arterial supply indicated persistent occlusion based on manual filling of the vasculature during necropsy. Therefore, it is possible that biochemistry results may not match tumor progression reliably. Alternatively, revascularization of the liver tumor could be achieved through collaterals acquired through adhesions from the omentum, gastrointestinal tract, body wall, or diaphragm. These possibilities were not evaluated at necropsy.

Tumor volume regression was variable and estimated to be approximately 12%–63%. Three patients evaluated by repeat CTA had persistent flow or recanalization of the HA through the thrombogenic coils in two dogs. These large tumors could have obtained blood supply from neighboring structures (e.g., omentum, gastrointestinal tract, body wall musculature, and diaphragm) and thus even when the HA is occluded, additional blood supply will remain in the largest, most exophytic liver tumors. This outcome could make complete dearterialization of the tumor difficult. A small case series involving four human patients indicated potential portal venous involvement in liver mass peripheral perfusion [21]. This finding also could contribute to subsequent tumor progression after dearterialization. Recanalization documented in the two dogs can be expected in circumstances when coil packing is insufficiently dense. Attempts to pack the coils tighter by using smaller more packable fibered coils, using glue or using plugs all could be considered in the future to decrease the probability of recanalization. A positive response to treatment was identified in these patients, which may be supported by the lack of recanalization. Cats received embolization beads in addition to the embolic coils. Beads were added in certain cases when all of the visualized blood supply went to the tumor, rather than the normal liver parenchyma, because more distal embolization is likely more effective although proximal embolization with coils is safer when performed along the entire primary hepatic arterial supply. The addition of the beads could contribute to complete, persistent, and more distal occlusion, but potentially it also may lead to more ischemic complications.

Animals survived at least 7 weeks postoperatively and no preoperative clinical signs or physical examination findings appeared to be contraindicated in pursuing this procedure. Two of the six patients with follow‐up examinations, one dog (Patient #1) and one cat (Patient #7), had abdominal effusions preoperatively and were reported to live to 119 and 505 days, respectively. These patients had resolution of the abdominal effusion approximately 2–3 weeks after dearterialization. This finding suggests a decrease in intra‐tumoral blood pressure once the arterial supply is removed that may decrease effusion in some of these cases.

Four patients were euthanized or died because of a disease process unrelated to the hepatic disease originally being managed. No initial clinical signs or physical examination findings were subjectively determined to negatively contribute to immediate procedural outcomes. Patients should be serially monitored for other concurrent disease processes that may be masked at the time of liver mass diagnosis.

Overall, we found that transarterial coil embolization of the HA could be an option as a palliative treatment of non‐resectable liver masses because all seven animals that received intra‐operative and postoperative antibiotics survived to discharge with minimal clinical signs of PES and no evidence of acute liver failure. Dearterialization of the liver will lead to transient increases in liver enzyme activities, which will improve over a 6–11 week period postoperatively. Changes in liver enzyme activities may not correlate with tumor response, and serial diagnostic imaging may be important to assess tumor response and success of embolization. Although this approach may be a novel palliative option for veterinary patients, its widescale adoption may be limited because of the requirements for fluoroscopy and highly specialized equipment. However, if further evaluation of this treatment option suggests patient benefit, this procedure could be performed using an open surgical approach as originally described.

## Disclosure

Authors declare no off‐label use of antimicrobials.

## Ethics Statement

Authors declare no Institutional Animal Care and Use Committee or other approval was needed. Authors declare human ethics approval was not needed.

## Conflicts of Interest

The authors declare no conflicts of interest.

## Supporting information


**Table S1.** Liver enzymes per patient in the preoperative, immediate postoperative, and the long‐term postoperative period.

**Table S2.** Complete blood count per patient in the preoperative, immediate postoperative, and the long‐term postoperative period.

**Table S3.** Patient signalment, diagnosis and cause of death.

**Table S4.** Patient comorbidities at time of procedure with number of coils used and tumor volume preoperatively and 6‐11 weeks postoperatively.
