# Supplementary material for: Hepatic Dearterialization for Nonresectable Liver Tumors in Five Dogs and Two Cats
Source: J Vet Intern Med. 2025 Mar 12;39(2):e70023. doi: 10.1111/jvim.70023 (PMC11898840; doi:10.1111/jvim.70023)
Supplement: Supplementary file 1 — Table S1. Liver enzymes per patient in the preoperative, immediate postoperative, and the long‐term postoperative period. [file JVIM-39-e70023-s002.docx]

| **Supplemental Table 1: Liver enzymes per patient in the pre-operative, immediate post-operative, and the long term post-operative period** | | | | | | | | | | | | | | | | | |
| --- | --- | --- | --- | --- | --- | --- | --- | --- | --- | --- | --- | --- | --- | --- | --- | --- | --- |
| **Patient** | **Pre-operative ALT (U/L)** | **Immediate post-operative ALT (U/L)** | **Long-term post-operative ALT (U/L)** | **Percent change pre-operative vs long-term post-operative ALT (%)** | **Pre-operative AST (U/L)** | **Immediate post-operative AST (U/L)** | **Long-term post-operative AST (U/L)** | **Percent change pre-operative vs long-term post-operative AST (%)** | **Pre-operative ALP (U/L)** | **Immediate post-operative ALP (U/L)** | **Long-term post-operative ALP (U/L)** | **Percent change pre-operative vs long-term post-operative ALP (%)** | **Pre-operative GGT (U/L)** | **Immediate post-operative GGT (U/L)** | **Long-term post-operative GGT (U/L)** | **Percent change pre-operative vs long-term post-operative GGT (%)** |  |
| 1 | 454 | 7,296 | 414 | -8.81 | 166 | 8,297 | 140 | -15.66 | 1,648 | 1,433 | 1,724 | 4.61 | 26 | 21 | 36 | 38.46 |  |
| 2 | 2,067 | 16,138 | - | - | 343 | 6,800 | - | - | 1,431 | 1,703 | - | - | 43 | 41 | - | - |  |
| 3 | 1,114 | 5,787 | 376 | -66.25 | 168 | 3,315 | 116 | -30.95 | 805 | 595 | 273 | -66.09 | 306 | 211 | 82 | -73.20 |  |
| 4* | 205 | 2,945 | 731 | 256.59 | 50 | 966 | - | - | 216 | 247 | 621 | 187.5 | 20 | 14 | 37 | 85.00 |  |
| 5 | 713 | 2,645 | 663 | -7.01 | 313 | 1,715 | 338 | 7.99 | 2,926 | 3,406 | 4,229 | 44.53 | 109 | 100 | 109 | 0.0 |  |
| 6 (cat) | 180 | 736 | 31 | -82.78 | 132 | 624 | 25 | -81.06 | 27 | 27 | 21 | -22.22 | 10 | 8 | 1 | -90.00 |  |
| 7 (cat) | 284 | 790 | 80 | -22.2 | 107 | 798 | 37 | -65.42 | 51 | 29 | 10 | -80.39 | 8 | 5 | 1 | -87.50 |  |
| *Patient diagnosed with metastatic stromal sarcoma and noted to be unwell with gastrointestinal clinical signs at the time of long-term post-operative repeat biochemistry | | | | | | | | | | | | | | | | | |

Abbreviations: ALT, alanine transaminase; AST, aspartate aminotransferase; ALP, alkaline phosphatase; GGT Gamma-glutamyl Transferase
