# Supplementary material for: Hepatic Dearterialization for Nonresectable Liver Tumors in Five Dogs and Two Cats
Source: J Vet Intern Med. 2025 Mar 12;39(2):e70023. doi: 10.1111/jvim.70023 (PMC11898840; doi:10.1111/jvim.70023)
Supplement: Supplementary file 2 — Table S2. Complete blood count per patient in the preoperative, immediate postoperative, and the long‐term postoperative period. [file JVIM-39-e70023-s001.docx]

| **Supplemental Table 2: Complete Blood Count per patient in the pre-operative, immediate post-operative, and the long term post-operative period** | | | | | | | | | | | | | | | |
| --- | --- | --- | --- | --- | --- | --- | --- | --- | --- | --- | --- | --- | --- | --- | --- |
| **Patient** | **Pre-operative HCT (%)** | **Immediate post-operative HCT (%)** | **Long-term post-operative HCT (%)** | **Pre-operative WBC (K/uL)** | **Immediate post-operative WBC (K/uL)** | **Long-term post-operative WBC (K/uL)** | **Pre-operative Platelets (K/uL)** | **Immediate post-operative Platelets (K/uL)** | **Long-term post-operative Platelets (K/uL)** | **Pre-operative Neutrophils (K/uL)** | **Immediate post-operative Neutrophils (K/uL)** | **Long-term post-operative Neutrophils (K/uL)** | **Pre-operative Lymphocytes (K/uL)** | **Immediate post-operative Lymphocytes (K/uL)** | **Long-term post-operative Lymphocytes (K/uL)** |
| 1 | 39.8 | 32.8 (L) | 51.6 | 10.7 | 17.2 | 19.7 | 320 | 646 (H) | 716 | 8.31 | 15.03 (H) | 16.55 | 1.68 | 0.757 (L) | 1.97 |
| 2 | 30.9 (L) | 25.0 (L) | - | 19.5 (H) | 25.6 (H) | - | 1173 (H) | 579 | - | 17.94 (H) | 24.06 (H) | - | 0.975 (L) | 0.768 (L) | - |
| 3* | 50.0 | 39.9 | 41 | 13.3 | 22.0 (H) | 12.8 | 456 | 194 | 120 | 9.68 | 20.02 (H) | 10.62 | 1.543 | 0.88 (L) | 1.28 |
| 4 | 46.1 | 37.2 (L) | - | 11.9 | 39.4 (H) | - | 1083 (H) | 798 | - | 7.97 | 33.88 (H) | - | 1.428 | 1.182 | - |
| 5 | 60.3 | 34.0 (L) | 42.5 | 7.6 | 12.4 | 29.4 (H) | 451 | 370 | 345 | 6.32 | 10.35 | 19.85 | 0.585 (L) | 0.521 (L) | 3.028 |
| 6 (cat) | 31.8 | 26.6 (L) | 33.9 | 12 | 18.9 | 9.2 | 233 | 226 | 385 | 9.62 | 16.88 (H) | 7.44 | 1.596 | 1.266 | 1.156 |
| 7 (cat)** | 14.4 (L) | 23.1(L) | 28.7 | 38.0 (H) | 32.0 (H) | 28.4 (H) | 308 | 149 | 149 | 33.74 (H) | 29.44 (H) | 26.13 (H) | 2.356 | 1.6 | 1.42 |
| *Long term post-operative bloodwork performed at 11 weeks post-operatively instead of 6-8 weeks post-operative **Required transfusion pre and post-operatively | | | | | | | | | | | | | | | |

Abbreviations: HCT, hematocrit; WBC, white blood cell count
