# Supplementary material for: Hepatic Dearterialization for Nonresectable Liver Tumors in Five Dogs and Two Cats
Source: J Vet Intern Med. 2025 Mar 12;39(2):e70023. doi: 10.1111/jvim.70023 (PMC11898840; doi:10.1111/jvim.70023)
Supplement: Supplementary file 3 — Table S3. Patient signalment, diagnosis and cause of death. [file JVIM-39-e70023-s003.docx]

| **Supplemental Table 3: Patient signalment, diagnosis and cause of death** | | | | |
| --- | --- | --- | --- | --- |
| **Patient** | **Signalment** | **Dx at time of procedure** | **Dx via necropsy** | **Reported cause of death** |
| 1 | 13yo FS Chihuahua Mix | Unknown liver mass (not sampled) | **HCC** | Unexpected death secondary to **Congestive Heart Failure** on Necropsy |
| 2 | 12yo FS Maltese Mix | **Hepatic adenoma** diagnosed on punch biopsy | No necropsy performed | Unmanaged **diabetic ketoacidosis** |
| 3 | 13yo MN Poodle Mix | **Hepatocellular carcinoma** diagnosed by incisional biopsy | **HCC/Hemangiosarcoma** | Euthanasia due to hemoabdomen secondary to **metastatic hemangiosarcoma** reported via necropsy |
| 4 | 9yo MN Hound | **Metastatic Stromal** Sarcoma diagnosed by incisional biopsy | No necropsy performed | Euthanasia due to suspected **metastatic stromal sarcoma** |
| 5 | 10yo FS Maltese Mix | Previously diagnosed **chronic bridging hepatitis** with new mass lesions (not sampled) | No necropsy performed | Euthanasia due to **deterioration**, unknown cause |
| 6 | 12yo MN Domestic longhair | **Chloangiocystadenoma** diagnosed by laparoscopic liver biopsy | **Cholangiocarcinoma** | Euthanasia due to **deterioration,** suspect secondary to hemoabdomen |
| 7 | 13yo MN Domestic shorthair | **Biliary cystadenoma** diagnosed via FNA | **Biliary cystadenoma** | Euthanasia due to **progressive azotemia secondary to pyelonephritis** **and metastatic carcinoma** reported via necropsy |

Abbreviations: FS, Female spayed; MN, Male neutered; FNA, fine needle aspirate; HCC, hepatocellular carcinoma
