# Supplementary material for: Hepatic Dearterialization for Nonresectable Liver Tumors in Five Dogs and Two Cats
Source: J Vet Intern Med. 2025 Mar 12;39(2):e70023. doi: 10.1111/jvim.70023 (PMC11898840; doi:10.1111/jvim.70023)
Supplement: Supplementary file 4 — Table S4. Patient comorbidities at time of procedure with number of coils used and tumor volume preoperatively and 6‐11 weeks postoperatively. [file JVIM-39-e70023-s004.docx]

| **Supplemental Table 4: Patient comorbidities at time of procedure with number of coils used and tumor volume pre-operatively and 6-11 week post operatively** | | | | | | |
| --- | --- | --- | --- | --- | --- | --- |
| **Patient** | **Systemic disease** | **Effusions at time of procedure** | **Number of coils used** | **Pre-operative tumor volume (mm^3)** | **Post-operative tumor volume (mm^3)** | **Percent tumor volume change** |
| 1 | 1. Myxomatous Mitral Valve Disease (MMVD) – ACVIM stage B1 2. Unilateral left pylectasia on abdominal ultrasound | Abdominal effusion secondary to suspected extralesional venous compression or tumor hypertension | 10 | 329,710 | 246,921 | -25% |
| 2 | 1. Uninvestigated heart murmur 2. Bilateral nephroliths on reported on diagnostic imaging 3. Diffuse hyperattenuating regions along spleen 4. Diabetes Mellitus | Abdominal effusion | 9 | 608,904 | - | - |
| 3 | 1. MMVD – ACVIM stage B2 2. Bilateral evidence of chronic renal changes on diagnostic imaging 3. Hyporexia and diarrhea | None | 6 | 395,801 | 504,000* | 27% |
| 4 | 1. Metastatic stromal sarcoma with multiple splenic nodules and lung nodules present | None | 16 | - | - | - |
| 5 | 1. Chronic bridging hepatitis | None | 10 | 199,690 | 290,021 | 45% |
| 6 | 1. Uninvestigated heart murmur 2. Bilateral evidence of chronic nephropathy on diagnostic imaging | None | 14 | 527,834 | 191,755 | -64% |
| 7 | 1. Restrictive cardiomyopathy 2. Unilateral right pylectasia reported on diagnostic imaging 3. Anemia requiring a blood transfusion 4. Melena | Bicavitary effusion suspect hemoabdomen secondary to hepatic mass and concurrent congestive heart failure | 5 | 541,080 | 474,586 | -12% |
| *Volume calculated on post-mortem exam | | | | | | |

Note: Tumor volumes were assessed using 3D slicer on a pre-operative and post-operative CT. The percent volume change is calculated on the difference between the pre-operative and post-operative 3D slicer volume calculations.

Abbreviations: MMVD, Myxomatous mitral valve disease
